# Impacts of the COVID-19 pandemic on elective cataract surgeries

**DOI:** 10.31744/einstein_journal/2022AO6687

**Published:** 2022-05-02

**Authors:** Marcelo Tannous, Renata Leonel Freire Mendes, Ana Vega Carreiro de Freitas, Andressa Miranda Magalhães, Rogério Ferrari, Bruno Luiz Miolo, Milton Ruiz Alves, Amaryllis Avakian, Pedro Carlos Carricondo

**Affiliations:** 1 Hospital das Clínicas Faculdade de Medicina Universidade de São Paulo São Paulo SP Brazil Hospital das Clínicas, Faculdade de Medicina, Universidade de São Paulo, São Paulo, SP, Brazil.

**Keywords:** Cataract, Phacoemulsification, Cataract extraction, COVID-19, Coronavirus infections

## Abstract

**Objective:**

To evaluate the standards of practice of Brazilian cataract surgeons in relation to the protective measures adopted to mitigate the risks of transmission of COVID-19 during cataract surgery, in asymptomatic patients.

**Methods:**

A descriptive, cross-sectional, quantitative paradigm study, developed from a self-administered electronic questionnaire sent to ophthalmologists and residents/specialists in ophthalmology in Brazil, who performed cataract surgeries in 2019 and 2020, connected through social media and mail listing from local societies.

**Results:**

Of the 303 participating surgeons, 159 (n=52.2%) performed elective cataract surgeries between March 20^th^, 2020 to June 1^st^, 2020. Among the measures adopted by ophthalmologists with the purpose of preventing viral transmission, the patient’s temperature was measured by 84.3% (n=134), and the verification of respiratory symptoms and contact/exposure to cases of COVID-19 by 87.4% (n=139). Most did not submit their patients to laboratory tests to detect COVID-19 (145; 91.2%). In surgery, 44.7% (n=71) used an N95 mask, and 69.2% (n=110) kept their patients with a mask. No stage of phacoemulsification was modified in 144 (90.6%) participants, 13 (8.2%) added methylcellulose under the main incision, and two (1.3%), modified another surgical stage.

**Conclusion:**

The COVID-19 pandemic significantly interrupted part of cataract surgeries in Brazil from March to June 2020 and measures to prevent viral spread are being heterogeneously adopted by surgeons. Understanding these measures could be the first step to improve strategies to return to pre-pandemic levels.

## INTRODUCTION

Since being declared a pandemic by the World Health Organization (WHO) on March 11^th^, 2020, the coronavirus 2019 disease (COVID-19) continues to affect increasing numbers of people worldwide.^(1)^ In response to the significant increase in demand for medical resources, regulatory bodies and boards have advised their members to delay all elective surgeries as much as possible during this period, including cataract surgeries.^(2,3)^ However, with the reduction of the transmission rate in some locations, an understanding of the nature of the disease, and easing of social distancing measures, elective surgeries have resumed, following authorization through state and local ordinances, which regulate resumption protocols.

Several medical entities, such as the Brazilian Council of Ophthalmology (CBO), the American Academy of Ophthalmology (AAO) and the National Health Surveillance Agency (ANVISA - *Agência Nacional de Vigilância Sanitária*), released documents with guidelines for the resumption of elective surgeries.^(2-4)^ Among the recommendations are the availability of appropriate COVID-19 tests, with the understanding that everyone is a possible carrier of the severe acute respiratory syndrome coronavirus 2 (SARS-CoV-2) if testing is not done; preoperative patient testing, depending on service availability; definition of strategies for referral of positive, and suspect COVID-19 workers and patients; and use of N95 masks by healthcare professionals performing aerosol-generating procedures, and use of surgical masks by patients.^(2-4)^ Despite the existence of the resumption protocols, it is still not known whether the teams are aware of these recommendations for the prevention of COVID-19 dissemination in elective surgeries and if they are being adopted.

## OBJECTIVE

To evaluate the standards of practice of Brazilian cataract surgeons in relation to the protective measures adopted to mitigate the risks of transmission of COVID-19 during cataract surgery, in asymptomatic patients.

## METHODS

A descriptive, cross-sectional, quantitative paradigm study, developed from a self-administered electronic questionnaire sent to ophthalmologists and ophthalmology residents/specialists in Brazil, who performed cataract surgery in 2019 and 2020.

Individuals of all ages and both sexes, who worked professionally in Brazil, were included. The research was disclosed to the reference population through the mailing lists of the Brazilian Laser Ophthalmologic Surgery Society and the Department of Ophthalmology at the *Universidade de São Paulo* (USP), and through social media (closed Facebook and WhatsApp groups of the specialty), between September and October 2020. In each invitation, an explanatory letter and the link to access the questionnaire and the Informed Consent Form were sent, in which the participants were informed about the research, the nature of the data, its objectives and possible risks, and voluntarily signed their participation. Subsequently, the records were analyzed using a structured script, built specifically to systematize the study of the data. Physicians who had not performed cataract surgery in the previously mentioned period, or did not fully complete the questionnaire for this study were excluded. The period for receiving responses was from July to September 2020.

To prepare the data collection instrument, exploratory research was used as a preliminary step, involving literature review in PubMed^®^ and Scopus databases, and using the descriptors “elective surgical procedures”, “COVID-19”, and “ophthalmology” “survey”, besides asynchronous discussions among the authors. The data from the self-administered questionnaire, containing 15 structured questions, included demographic variables (age, sex, and professional practice status), and those related to the impact of the pandemic on the performance of surgeries (prior surgical volume, surgical volume between March and June 2020, and time of suspension of cataract surgeries). In addition, there were questions about the measures adopted to mitigate viral transmission in cataract surgeries, involving patient screening (checking temperature, performance of reverse transcriptase polymerase chain reaction - RT-PCR -, serology or rapid test, verification of respiratory symptoms, and exposure to sick people), surgical technique (changes in technique and use of adhesive field), and use of masks (use of N95 by the physician and use of mask by the patient).

Quantitative variables were described using ranges of values. Categorical variables were described using frequencies and percentages.

The study was developed in accordance with the guidelines and standards regulating research involving human beings from Resolution 466 of 2012 of the National Health Council, and obtained approval from the Research Ethics Committee of the *Hospital das Clínicas da Faculdade de Medicina da USP* before its performance, under opinion # 4.250.225 and CAAE: 36796620.0.000.0068.

## RESULTS

### Overall demographics and surgical volume of cataract surgeons

A total of 303 surgeons participated in the study. Of these, 76.2% (n=231) were male, with a predominance of the 30-39-year-old age group (45.2%, n=137). Regarding the region of practice in Brazil, there was a prevalence of the Southeast Region (n=178, 58.7%), more specifically the state of São Paulo (n=143; 40.6%), as shown on table 1.

**Table 1:** Overall demographics and surgical volume of cataract surgeons

Characteristics	n (%)
Sex	
Male	231 (76.2)
Female	72 (23.8)
Age range, years	
20-29	30 (9.9)
30-39	137 (45.2)
40-49	87 (28.7)
50-59	30 (9.9)
59-69	15 (5.0)
Over 70	4 (1.3)
Region of practice	
Southeast	178 (58.7)
Northeast	57 (18.8)
South	46 (15.1)
North	24 (7.9)
Midwest	18 (5.9)

Regarding surgical volume in 2019, most surgeons (n=114; 37.6%) said they performed 100 to 300 cases per year (nine to 25 cases per month) (Table 2).

**Table 2:** Prior surgical volume, suspension time, and performance of surgeries during the pandemic

Surgical volume	n (%)
Number of surgeries in 2019	
>500	74 (24.4)
301-500	47 (15.5)
100-300	114 (37.6)
<100	68 (22.5)
Total suspension time of surgeries after the start of the pandemic, months	
>3	76 (25.1)
2-3	67 (22.1)
1-2	91 (30.0)
No suspension	10 (3.3)
Performance of surgeries from March to June 2020	
Yes	159 (52.2)
No	144 (47.8)

Regarding the suspension of elective cataract surgeries from the beginning of the pandemic until October 2020, most participants (n=91; 30%) reported total interruption of activities for a period of 1 to 2 months. More specifically at the beginning of the pandemic (between March 20^th^ and June 1^st^), more than half of the physicians (52.2%, n=159) performed elective cataract surgeries.

### Demographics and surgical volume of cataract surgeons who performed procedures during the period of March 20**th** to June 1**st**, 2020

Of the 159 professionals who performed surgeries during the beginning of the pandemic in Brazil (Table 3), 127 (79.9%) were male.

**Table 3:** Overall demographics and surgical volume of cataract surgeons (n=159 participants) who performed surgery from March to June 2020

Characteristics	n (%)
Sex	
Male	127 (79.9)
Female	32 (20.1)
Age range, years	
20-29	16 (10.0)
30-39	64 (40.2)
40-49	46 (28.8)
50-59	25 (15.7)
59-69	8 (5.0)
Region of practice	
Southeast	77 (48.4)
Northeast	22 (13.8)
South	37 (23.3)
North	9 (5.7)
Midwest	14 (8.8)

The majority were in the 30-39-year-old age group (n=64; 40.2%), followed by the 40-49-year-old age group (n=46; 28.8%). The predominant region of professional practice was the Southeast (n=77; 48.4%), especially the State of São Paulo (n=52; 32.7%). The surgical volume in 2019 was greater than 500 cases for 34.6% (n=55) of them, from 100 to 300 cases for 32.1% (n=51), from 301 to 500 cases for 18.2% (n=29), and less than 100 cases for 15.1% (n=24) of physicians.

### Measures to prevent the spread of COVID-19 in cataract surgeries

#### Screening for COVID-19

**Figure 1:**
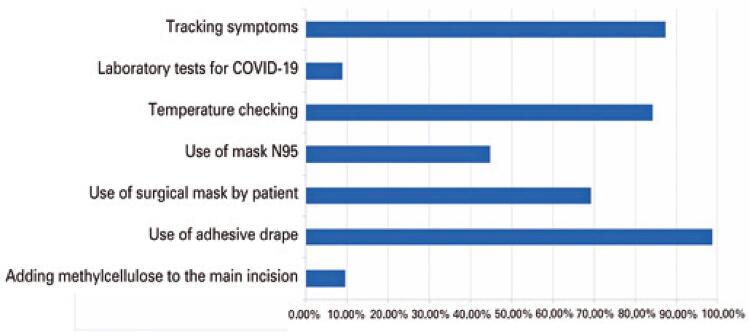
Main measures adopted by cataract surgeons to mitigate the risks of viral transmission during the pandemic of COVID-19 (n=159)

As to the measures adopted by surgeons who performed cataract surgery during the beginning of the pandemic (n=159), temperature measurement of the patient and surgical team was the procedure most performed (45.3%, n=72). Questioning about respiratory symptoms and patient and staff contact/exposure to cases of COVID-19 occurred in 40.9% (n=65) of cases (Table 4) (Figure 1).

**Table 4:** Screening measures adopted by cataract surgeons to mitigate the risks of viral transmission during the COVID-19 pandemic

	n (%)
Temperature check	
Of the patient and of the team	72 (45.3)
Only the patient	62 (39.0)
Do not check temperature	25 (15.7)
Questioning as to respiratory symptoms and contact/ exposure	
Of the patient and of the team	65 (40.9)
Only of the patient	74 (46.5)
Only of the team	1 (0.6)
Did not question	19 (11.9)
Performance of laboratory tests	
Did not do	145 (91.2)
RT-PCR	6 (3.8)
Rapid test	5 (3.1)
Serology	3 (1.9)
Time of laboratory tests, days before surgery	
1-3	13 (92.8)
7	1 (7.2)

RT-PCR: reverse transcriptase polymerase chain reaction.

Most study participants did not submit their patients to laboratory tests (RT-PCR, serology, and rapid test) to detect COVID-19 (n=145; 91.2%) preoperatively. Of these, 92.8% (n=13) ordered the test one to 3 days before surgery and 7.2% (n=1), one week before.

#### Use of masks

The use of N95 masks during cataract surgeries was reported by 44.7% (n=71) of the participants (Table 5) (Figure 1).

**Table 5:** Measures taken by cataract surgeons during surgery to mitigate the risks of viral transmission during the COVID-19 pandemic

	n (%)
Mask used by the surgeon	
N95	71 (44.7)
Surgical mask	88 (55.3)
Use of a mask by the patient	
Yes	110 (69.2)
No	49 (30.8)
Use of adhesive drape	
Yes	157 (98.7)
No	2 (1.3)
Modification in the phacoemulsification technique	
None	144 (90.6)
Addition of methylcellulose to the main incision	13 (8.2)
Modification of another surgical stage	2 (1.3)
Already added methylcellulose to the incision before the pandemic	2 (1.3)

#### Surgical technique

Most ophthalmologists (n=157; 98.7%) reported using an adhesive drape on the patient during cataract surgery. For phacoemulsification, 144 (90.6%) of the 159 ophthalmologists stated not having modified any surgical step to reduce the spread of aerosols (Table 5).

## DISCUSSION

In an effort to reduce SARS-CoV-2 transmission, many ophthalmologic societies, including the AAO and CBO, recommended immediate suspension of any non-emergency and emergency treatments for a minimum of 4 weeks (beginning March 18^th^, 2020), which extended through July 2020.^(2,3)^ Although the scientific basis for estimating the risk of SARS-CoV-2 infection during most eye surgeries is at an early and evolving stage, most patients awaiting cataract surgeries are elderly with comorbidities, and at higher risk of COVID-19-related mortality. The cancellation of elective cataract surgeries, the most commonly performed procedure in ophthalmology, would thus outweigh the benefits that the operation could bring to patients at that time.

With the end of the emergency status declared by most countries, the AAO began to advocate the prioritization of semi-urgent cases. Cataract surgery is considered semi-urgent or non-elective when the affected individual has great functional impairment, such as inability to drive, work, or practice self-care; there is also a great risk of falling, and/or cases of intolerable anisometropia, phacomorphic and phacolytic glaucoma, and penetrating ocular trauma, which justify surgery within days to weeks.^(2)^ More recently, it is recommended to prioritize essential elective surgeries, defined as those with 3 to 8 weeks to be performed, and the entire surgical program should be reviewed regarding its risks, priorities, and resources.^(4)^

In the current study, more than half of physicians (n=159; 52.2%) performed elective cataract operations between March 20^th^ and June 1^st^, 2020. Conversely, a survey conducted by the European Society of Cataract Surgeons and Refractive Surgery (ESCRS) of 1,376 physicians, in April 2020, predominantly from Europe, found that almost 60% of surgeons had completely discontinued their operative activities.^(5)^ In India, in a study conducted in the same period with 347 professionals, only 5.7% (n=20) still performed elective cataract surgery, prioritizing mainly emergency eye surgery.^(6)^Another study in India noted a 99.7% decrease in surgical volume from March 25 to May 3, 2020, compared to the previous year.^(7)^

For ophthalmic procedures involving close physician-patient proximity, N95 or similar masks provide better protection for the ophthalmologist, since they have a minimum filtration efficacy of 95%.^(3)^However, due to the increased demand in recent months, these masks may be more restricted and difficult to access in some regions.^(3)^ Surgical masks also dramatically reduce the transmission of respiratory viruses from an infected individual, including SARS-CoV-2, and are considered sufficient for the surgeon in most cases.^(2)^ The AAO and ANVISA have also advised patients wear a surgical mask during any ophthalmologic procedure, circulation in the operating room, and hospitalization after the procedure, to prevent asymptomatic transmission to the surgeon and staff.^(2,4)^ In the present study, most of the surgeons (n=71; 44.7%) used N95 masks during the surgeries, and most of them kept their patients with masks on during the surgery (69.2%, n=110).

Regarding preoperative laboratory tests, their role is controversial and their accuracy, availability, and practicality are taken into consideration for the rules of each individual institution.^(2)^ In the present study, accordingly, 9.8% (n=14) of physicians submitted their patients to laboratory tests for COVID-19. In India, of 1,260 ophthalmologists interviewed, 9.9% (n=124) ordered preoperative screening laboratory tests.^(6)^ As a recommendation, the AAO and the American College of Surgery (ACS) advise that for all elective surgical procedures that may generate aerosols (such as in cases of general anesthesia), RT-PCR should be performed on asymptomatic patients.^(2,8)^ If the patient has not had the test, the AAO indicates the use of the N95 mask together with eye protection for all operating room staff.^(2)^ More recently, ANVISA recommended in its Technical Note that, for all services with available resources, real-time RT-PCR should be performed.^(4)^ If the patient is symptomatic or has a positive COVID-19 test, a surgical mask, face shield or goggles, apron, and gloves are sufficient for situations with no risk of aerosolization with infectious particles.^(4)^ In procedures with risk of aerosolization, the use of a N95/PFF2 mask or equivalent by the team is added.^(4)^

Some guidelines recommend pre-screening of remote surgical patients (telephone contact, for example) to verify respiratory symptoms and possible close contact with cases of COVID-19, as well as checking the temperature before entering the operating room.^(2,4,9)^ The health status of all members of the surgical and anesthetic team should also be considered, with removal of these professionals in case of fever or any respiratory symptoms.^(4,10)^ This questioning was made to patients by most participants in this study (n=139; 87.4%). However, only 40.9% (n=65) questioned staff and patient simultaneously, which may have relevance especially regarding the employees who are not health professionals and would not be aware of the possible symptoms of the disease. Temperature measurement in the service was referred by 84.3% (n=134) of the interviewed physicians, with less than half of them simultaneously measuring the temperature of the patient and the staff (n=72; 45.3%).

For cases of patients exposed to SARS-CoV-2, Anvisa recommended that elective procedures should be postponed for at least 14 days. In asymptomatic or mildly symptomatic positive cases, for 4 weeks; in symptomatic positives without hospitalization, 6 weeks; in hospitalized positive or diabetic/immunocompromised, 8 to 10 weeks, and in positive patients who were admitted to an intensive care unit, surgery should be postponed for 12 weeks.^(4)^

This study also found that most surgeons did not change the phacoemulsification technique (n=144; 90.6%), and some started adding methylcellulose under the main incision (n=13; 8.2%) - both actions consistent with current studies. Although SARS-CoV-2 has been isolated on the conjunctival surface and other viruses have been isolated in the aqueous humor, it is not known at this time whether it is present in the anterior chamber.^(11-13)^The potential generation of aerosols and visible microdroplets by phacoemulsification surgery, in turn, was first suggested by McGhee et al. and by Darcy et al., in laboratory, with animal and human eyes.^(14,15)^ However, this effect was not observed with the use of a 2.2mm microincision and coating of the corneal surface with 2% hydroxypropyl methylcellulose (HPMC) at the main incision for a period of approximately 67 seconds.^(15)^ Other studies also did not show aerosol generation in the laboratory^(16-18)^ and in living patients.^(19)^ Furthermore, the phacoemulsification procedure starts with the replacement of aqueous humor with viscoelastic, which is then replaced by balanced salt solution from the phaco tip.^(2)^ Even if aerosolization were to occur, it would be from the balanced salt solution, and not from the patient’s aqueous humor.^(2)^ Additionally, povidone-iodine solutions, which have high virucidal power against a wide range of viruses, including severe acute respiratory syndrome coronavirus 1 (SARS-CoV-1) and Middle East respiratory syndrome (MERS-CoV), are routinely used in preoperative antisepsis and may be active against SARS-CoV-2.^(20,21)^ Considering these findings, the risk of aerosolized virus during surgery is very low.^(2)^

Currently, with the advent of vaccination, although not present in the questionnaire since it was not the reality at that time, it is suggested to wait 7 days between the vaccination and the surgical procedure, to avoid fusion regarding possible complications or vaccine reactions, in case symptoms appear in the patient. Likewise, there is no minimum interval for vaccination after surgeries.^(4,22-24)^It is also recommended to reduce as much as possible the number of people inside the operating room and to train the professionals involved as to the signs and symptoms of COVID-19, safe use of Personal Protective Equipment (PPE), hand hygiene, and other guidelines to prevent contamination, besides prioritizing telehealth/telemedicine resources.^(4)^

### Study limitations

As a limitation, we emphasize that the instrument evaluated ophthalmologists from a continental country such as Brazil, in which the COVID-19 pandemic broke out at different times due to demographic differences among regions. Because this was a cross-sectional study, the precautionary patterns against coronavirus transmission adopted by ophthalmologists who were at different moments of the same pandemic were evaluated. This pattern may have changed over the weeks as new government regulations were imposed, new protective measures were discovered, or because they became more popular and affordable. The number of surgeons who responded to this survey limits the generalization of the study, and does not represent the full ophthalmic fraternity, considering that Brazil has 20,455 ophthalmologists.

## CONCLUSION

The COVID-19 pandemic led to a significant interruption of part of cataract surgeries in Brazil, and measures to prevent the spread of SARS-CoV-2 are being adopted heterogeneously by Brazilian cataract surgeons. Most of the questions asked in this study did not appear in other surveys in the literature, and allowed us to delineate patterns of mask use by patients, preoperative epidemiological screening for COVID-19, and modifications in surgical technique pattern to prevent aerosol dissemination.

COVID-19 and strategies for preventing the disease by asymptomatic patients in elective surgeries are still challenges for the scientific community at large. It is possible that more evidence will emerge in the coming days that will change the guidelines on measures to limit its spread. Understanding the measures already taken by ophthalmologists may be the first step in improving these strategies, which are essential to returning elective eye procedures to the levels of care they were able to provide to patients before the pandemic.
